# Sporadic Creutzfeldt-Jakob disease prion infection of human cerebral organoids

**DOI:** 10.1186/s40478-019-0742-2

**Published:** 2019-06-14

**Authors:** Bradley R. Groveman, Simote T. Foliaki, Christina D. Orru, Gianluigi Zanusso, James A. Carroll, Brent Race, Cathryn L. Haigh

**Affiliations:** 1 Division of Intramural Research, Laboratory of Persistent Viral Diseases, National Institute of Allergy and Infectious Diseases, Rocky Mountain Laboratories, National Institutes of Health, 903 South 4th Street, Hamilton, MT 59840 USA; 2 Department of Neurosciences, Biomedicine and Movement Sciences, University of Verona, Verona, Italy

**Keywords:** Prion, CJD, Human cerebral organoid, Induced pluripotent stem cells, RT-QuIC

## Abstract

For the transmissible, neurogenerative family of prion diseases, few human models of infection exist and none represent structured neuronal tissue. Human cerebral organoids are self-organizing, three-dimensional brain tissues that can be grown from induced pluripotent stem cells. Organoids can model aspects of neurodegeneration in Alzheimer’s Disease and Down’s Syndrome, reproducing tau hyperphosphorylation and amyloid plaque pathology. To determine whether organoids could be used to reproduce human prion infection and pathogenesis, we inoculated organoids with two sporadic Creutzfeldt-Jakob Disease prion subtypes. Organoids showed uptake, followed by clearance, of the infectious inoculum. Subsequent re-emergence of prion self-seeding activity indicated de novo propagation. Organoid health assays, prion titer, prion protein electrophoretic mobility and immunohistochemistry demonstrated inoculum-specific differences. Our study shows, for the first time, that cerebral organoids can model aspects of human prion disease and thus offer a powerful system for investigating different human prion subtype pathologies and testing putative therapeutics.

**Electronic supplementary material:**

The online version of this article (10.1186/s40478-019-0742-2) contains supplementary material, which is available to authorized users.

## Introduction

The transmissible, neurodegenerative prion diseases affect humans and animals. Acquired prion diseases arise due to exposure to prion contaminated material. However, prion diseases more often occur sporadically or due to hereditary mutations within the gene encoding the prion protein (PrP). In cases of human sporadic Creutzfeldt-Jakob Disease (sCJD), different molecular subtypes of prions have been recognized [4, 13, 16, 32, 33] that influence the clinical and pathological disease phenotypes. These subtypes are identified by the electrophoretic mobility pattern of the disease-associated, protease-resistant prion protein (PrP^Res^) and by a genetic polymorphism at amino acid residue 129, which may be a methionine or valine. The heterogeneity of the human subtypes and the brain regions they target [39] causes complications when attempting to model human diseases in cell models as homogenous cell systems cannot address different neuronal vulnerabilities.

There are further challenges associated with investigating human prion diseases in vitro. First and foremost, human cell lines have traditionally proven refractive to prion infection. Only two cell models of prion infection and propagation have been described that express normal human PrP levels in a human cellular background. The first, published in 1995, reported stable CJD infection in sub-clones from the Sh-SY5Y neuroblastoma cell line [19] but no further use of this model has ever been reported. Recently, the second showed that astrocytes differentiated from human induced pluripotent stem cells (hu-iPSCs) can take up and propagate prion infection from sCJD samples [18]. Uptake of infection by these astrocytes was influenced by sCJD subtype and residue 129 polymorphism (M or V) of both the host cells and the inocula. This is the first fully human cell model developed since the 1995 Sh-SY5Y study, and the only human model of infection currently reported. Despite the significant advancement this model offers, it does not allow for studying how human neurons respond to infection or for investigating potential three-dimensional cellular cross-talk.

Hu-iPSCs have also been used to produce three-dimensional, highly organized cerebral tissues; referred to as cerebral organoids [20, 35]. Cerebral organoids display self-organization that generates an organ-like configuration and, following the development of cortex neuronal populations, also populate with astrocytes and oligodendrocytes creating the closest in vitro model of human brain tissue yet described [20, 25, 35]. Organoids also develop functional electrical signaling and can survive in culture for many months; some may survive years [20, 44]. Cerebral organoids have demonstrated substantial potential for modelling developmental diseases, such as the microcephaly associated with Zika virus infection [21, 38]. Plus, their longevity permits their use for investigating brain diseases that develop over a longer time frame. For example, organoids exhibit the tau hyperphosphorylation plus amyloid deposition associated with Alzheimer’s Disease and Down’s syndrome dementia [14].

Organoids or neuronal cultures from hu-iPSCs have similarly been used to model prion dysfunction in cells from people who are predisposed to developing prion disease due to mutations within the prion gene [14, 26]. Organoids generated from a patient carrying a Y218N PrP mutation (that causes Gerstmann-Sträussler-Scheinker prion disease) were able to reproduce certain pathological changes, such as astrogliosis and tau hyperphosphorylation, but did not replicate PrP^Res^ production [26]. We hypothesized that, in addition to their use modeling genetic prion diseases, cerebral organoid cultures could be used to develop an in vitro human model of prion infection in organized neuronal tissue by exposing them to sCJD prions. In this study we found that human cerebral organoids are susceptible to prion infection. Uptake of infection was influenced by the infecting inocula and the de novo prions produced were influenced by the underlying organoid. We conclude that human cerebral organoids offer a new three-dimensional, neuronal tissue model of human prion disease.

## Materials and methods

### Human induced pluripotent stem cells and culture

KYOU-DXR0109B (ACS-1023; ATCC) hu-iPSCs were routinely cultured on low growth factor Matrigel (Roche) in mTeSR1 medium (Stem Cell Technologies) with 5% CO_2_ in a humidified incubator as described in the mTeSR handbook. Colonies were passaged at approximately 70–80% confluency before colonies had started to contact each other.

### Human cerebral organoid generation and routine culture

Cerebral organoids were generated as described in Lancaster and Knoblich [20]. The following variations to the protocol were made: For embryoid body (EB) production one 25 cm^2^ flask of hu-iPSCs was used to seed a 96-well plate. Cells were plated in 100 μl hES medium (20% [v/v] knock-out serum replacement, 0.03% [v/v] fetal bovine serum, 1× glutamax, 1× non-essential amino acids, and 1 μl / 142.86 ml 2-Merceptoethanol in DME-F12 medium, additionally supplemented on day 0 with 50 μM Y27632 and 4 ng/ml rh FGF), with a further 100 μl added at days 2 and 4. EBs were transferred into neural induction medium (1× glutamax, 1× non-essential amino acids, 1% [v/v] N2, and 1 μg/ml heparin in DME-F12 medium) in low adhesion plates between days 4 and 6 depending upon morphology. Organoids were embedded 2–4 days after neural induction when neuroecotoderm was visible. Matrigel embedding was done in a 6-well plate with 6 organoids per well using 30 μl of Matrigel (Roche) and incubated in cerebral organoid media (1× glutamax, 1× penicillin-streptomycin solution, 0.5× non-essential amino acids, 0.5% [v/v] N2, 1 μl/4 ml insulin, and 1 μl/286 ml 2-Merceptoethanol in 1:1 Neurobasal:DME-F12 medium) with 1% (v/v) B12 minus retinoic acid. On day four, organoids were transferred into cerebral organoid medium with 1% (v/v) B12 plus retinoic acid for long-term culture. Long-term agitated culture was performed in upright 25 cm^2^ low-adhesion flasks on an orbital shaker at 85 rpm. Media was changed twice weekly.

### Prion infections of human cerebral organoids

Brain homogenates from sporadic CJD subtypes MV1 and MV2 were diluted into organoid maintenance media to a final concentration of 0.1% (tissue wet weight/volume). The inoculated media was filter sterilized through a 0.22 nitrocellulose filter. To ensure the infectious seeds within the inoculum had not been lost in the filtering process, RT-QuIC analysis (described below) was performed on the media. Significant RT-QuIC seeding activity was found in both MV1 and MV2 inocula (Table 1). Organoids were distributed between 6-well plates (3 organoids per well) for Prestoblue and LDH monitoring and T25 flasks for routine maintenance (6–12 organoids/flask). At the start of infection, existing media was removed from the organoids and replaced with the inoculated media. Twenty-four hours after inoculation an equivalent volume of fresh media was added to the cultures (diluting the original inoculum 1 in 2). Organoids were incubated for a further 3 days before a half media exchange. A full media and culture vessel exchange was performed 7 days after initial exposure. Organoids were maintained in agitated culture with three media changes per week. All brain tissues used in this study were obtained on autopsy and were therefore exempt from review by the NIH Office of Human Subjects Research Protections.

**Table 1:** sCJD inoculum information

Inocula	Gender	Age at onset (years)	Disease duration (months)	Inoculum SD_50_/μl
MV1	M	62	14	1.58 × 10^5^
MV2	M	57	15	8.89 × 10^5^

### Prestoblue analysis

Prestoblue metabolism was measured as per the manufacturer’s instructions once weekly or as required (the same 3 organoids were monitored from start to finish of the cultures). Briefly, Prestoblue reagent was diluted 1 in 10 in organoid media. Existing organoid media was removed and organoids were incubated in Prestoblue-containing media for 30 mins. The metabolized Prestoblue containing media was then transferred into replicate wells for analysis. Prestoblue fluorescence was measured at 560 nm excitation and 590 nm emission in a ClarioStar plate reader (BMG).

### Lactate Dyhydrogenase (LDH) analysis

Extracellular LDH was measured using cytotoxicity detection kit plus [LDH] (Roche). Results shown are obtained from the same 3 organoids as used for Prestoblue analysis. Organoids in flasks were occasionally assayed to ensure consistency with the plates (data not shown). Prior to the assay start the dye solution and catalyst solution were mixed 45:1. One hundred microliters of test or control medium, 24 h following a complete media change, was assayed in a 96-well tissue culture plate in triplicate by adding 100 μl of the dye-catalyst solution and incubating for 30 mins at room temperature. The reactions were terminated by addition of 50 μl of stop solution. The plate was mixed in a ClarioSTAR plate reader (BMG) for 10 s before reading absorbance at 492 nm.

### Immunofluorescence

Organoids were fixed and immuno-stained as described previously [9]. FoxG1 (Abcam) and GFAP (Abcam) primary antibodies were used at a 1 in 50 dilution. Secondary Alexafluor 488 or 555 antibodies (Invitrogen) were applied at a 1 in 250 dilution and organoids were mounted in Fluoromount medium (ThermoFisher) with curing at room temperature for 24 h.

### Histochemistry

Antigen retrieval was performed as previously described [34], followed by staining using the anti-prion monoclonal antibody 6H4 (Prionics). Astrocyte detection was performed by staining with polyclonal rabbit anti-glial fibrillary acidic protein (anti-GFAP; Dako). Slides were also stained for observation of overall pathology using a standard hematoxylin-eosin (H&E) protocol. All histopathology slides were analyzed by observers blinded to the inoculation groups using Aperio Imagescope software.

### RT-QuIC

Real-time QuIC (RT-QuIC) assays were performed similarly to those reported previously [30, 31]. Briefly, the RT-QuIC reaction mix contained 10 mM phosphate buffer (pH 7.4), 300 mM NaCl, 0.1 mg/ml hamster recombinant PrP 90–231 (purified as described in [31]), 10 μM thioflavin T (ThT), and 1 mM ethylenediaminetetraacetic acid tetrasodium salt (EDTA). Reaction mixes for culture media seeds (from the initial inoculum and samples collected throughout incubation) contained an additional 0.002% SDS in the reaction mix. Organoids were homogenized by motorized pestle to 10% (w/v) in PBS and cleared with a 2000×g 2 min centrifugation. Organoid homogenates were serially diluted in 0.1% SDS/PBS/N2 solution for a final SDS concentration of 0.002% in the reaction mix. For media seeded and organoid seeded reactions, respectively, either 80 or 98 μl of reaction mix was loaded into a black 96-well plate with a clear bottom (Nunc), and reaction mixtures were seeded with 20 μl of media or 2 μl of the indicated dilution of organoid homogenate for a final reaction volume of 100 μl and the same final concentrations in the reaction mix as indicated above. Plates were sealed (Nalgene Nunc International sealer) and incubated in a BMG FLUOstar Omega plate reader at 50 °C for 50 to 120 h with cycles of 60 s of shaking (700 rpm, double-orbital) and 60 s of rest throughout the incubation. ThT fluorescence measurements (excitation, 450 ± 10 nm; emission, 480 ± 10 nm [bottom read]) were taken every 45 min. Spearman-Kärber analyses [11] was used to provide estimates of the concentrations of seeding activity units giving positive reactions in 50% of replicate reactions, i.e., the 50% “seeding doses” or SD_50_’s as previously described [42].

### Proteinase-K digests and Western blotting

10% organoid homogenates were treated with 5 μg/ml Proteinase K in 1% Sarkosyl for 1 h at 37 °C with 400 rpm shaking. The reactions were stopped by incubation with 1 μM Pefabloc for 5 min at 4 °C. Samples were then mixed 1:1 with 2X Bolt LDS sample buffer (Invitrogen) containing 8% β-mercaptoethanol and boiled for 10 min. Samples were run on Bolt 4–12% Bis-Tris gels (Invitrogen) and transferred to PVDF membranes using the iBlot 2 transfer system (Invitrogen). PrP was detected using the 3F4 antibody (Millipore) at a 1:10,000 dilution and visualized using ECL Select (Amersham) and imaged on the iBright imaging system (Invitrogen).

### Cytokine arrays

A pool of conditioned media from flasks containing 6 organoids (media from the same 6 organoids was collected throughout) was collected at various time points during the incubation period. Media was assayed for cytokine levels using Bio-Plex Pro Human Inflammation Panel 1, 37-Plex (Biorad) as per the manufacturer’s instructions. Cytokines below the level of detection throughout the experiment are excluded from the analysis.

### Data analysis

Positive pixel counts were carried out using ImageScope v11.1.2.760 imaging software (Aperio). Statistical analysis, as indicated in figure legends, was carried out in GraphPad Prism 7.04. Z-scores, heatmap, cluster analysis, and dendrogram of the cytokine protein secretion by cerebral organoids were produced using the online application Heatmapper (http://www2.heatmapper.ca/) developed in the Wishart Research Group at the University of Alberta [2]. The clustering method chosen was the Average Linkage setting, and the dendrogram distance measurement method chosen was the Pearson setting, which establishes the distance as the absolute value of the Pearson correlation coefficient (between 0 and 1).

## Results

### Organoid infections

**Fig. 1:**
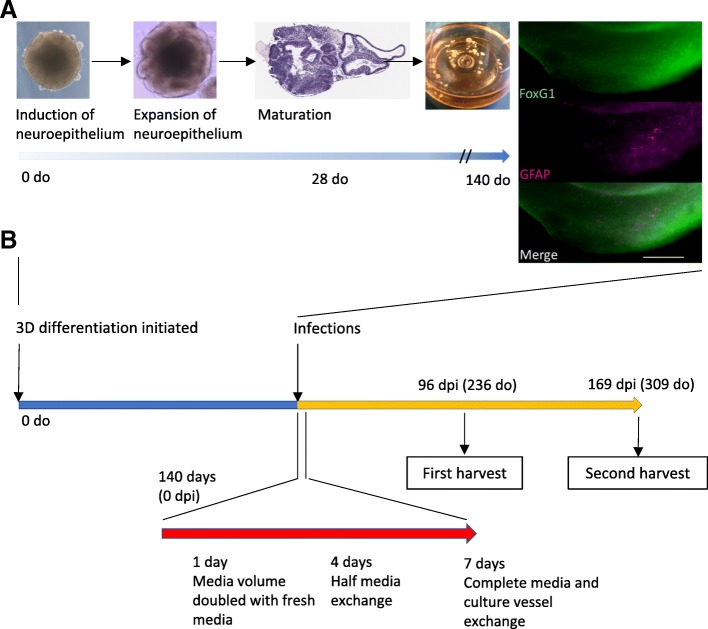
Developmental and experimental schematic*.* **a** Example phase images show organoids at early stages of development, becoming more structured, and organoid appearance as balls of tissue in an Erlenmeyer culture flask. H&E staining at 28 days old (do) shows the complexity of the organoids with many varied, structured domains. Immunofluorescence at 140 do confirms cortical identity (FoxG1) and shows that astrocytes (GFAP) have begun populating the neuronal layers. Scale bars = 200 μm. **b** Schematic time line of organoid maturation, infection and sampling

Cerebral organoids develop mature astrocytic and oligodendrocytic populations from ~ 2 months, and these continue to increase in number to ~ 5 months with all organoids displaying some astrocytes by ~ 140 days [35]. Astrocytosis is prominent in prion diseases [22] and astrocytes have been shown to accumulate disease-associated PrP prior to neuropathological changes [5, 10]. Therefore, we hypothesized that these cells may be important for the establishment of prion infection or the development of disease pathology. Human cerebral organoids were generated from induced pluripotent stem cells (iPSCs) [20]. To allow the cultures to develop glial populations, organoids were grown for 5 months (140 days) before commencing infections (organoid differentiation is shown in Fig. 1a with the timeline of infections in 1B). As previously mentioned, human prion disease phenotype and susceptibility are influenced by the methionine/valine polymorphism at amino acid position 129. The iPSCs used were heterozygous (129 M/V) for this polymorphism. To ensure optimal opportunity for uptake of infection by the organoids, we used brain homogenates from two sCJD patients who were also heterozygous at codon 129. In these sporadic cases, Proteinase-K resistant PrP had previously been glycotyped as type 1 or 2 CJD (nomenclature as described in [32]) and are referred to throughout as MV1 and MV2 accordingly. The MV1 and MV2 subtypes vary in disease duration and prevalence, with the MV1 subtype showing a generally faster disease progression but the MV2 subtype being ~ 3 times more common than the MV1 [17, 33, 36]. Our inocula were from cases with a similar disease duration and age of onset (Table 1).

### Organoids exhibited inoculum-related changes in health

**Fig. 2:**
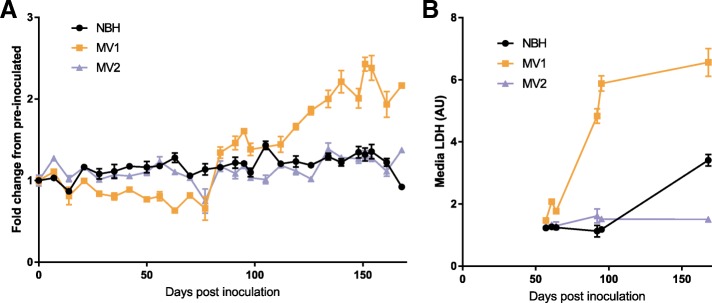
Organoid health and viability monitoring*.* **a** Prestoblue fluorescence, indicating changes in metabolism, expressed relative to pre-inoculated organoid metabolism. The same three organoids were monitored weekly throughout. **b** LDH detection in organoid culture media, indicative of cell death or loss of membrane integrity, of the same 3 organoids as in A. Data points shown mean and SD of 3 replicate reads

From the start of the infections, organoids were monitored for changes in their health using Prestoblue, a non-toxic probe that produces a fluorometric/colorimetric change within culture media in response to cell metabolism, including mitochondrial metabolism and enzymes involved in NADH/NADPH reactions. The MV1 inoculated organoids initially appeared to show slowing metabolism, but, 84 days post-infection (dpi), an almost two-fold increase was measured (Fig. 2a). This increase remained consistent for several weeks and then continued to climb until it fluctuated in the final weeks of the study. The MV2 inoculated organoids showed no change. At 92 dpi increased LDH levels, indicative of cell lysis and death, were detected in the culture media of the MV1 inoculated organoids (but not the NBH or MV2; Fig. 2b). Therefore, three organoids per condition were harvested for IHC analysis at 96 dpi. The remaining organoids continued to be monitored by Prestoblue metabolism and periodic LDH. At 168 dpi the NBH exposed organoids showed an increase in LDH and a decrease in Prestoblue. This could have been indicative of the organoids starting to die from aging and, therefore, the remaining organoids were harvested for IHC or protein analysis at 169 dpi.

### Infected organoids were positive for RT-QuIC seeding activity

**Fig. 3:**
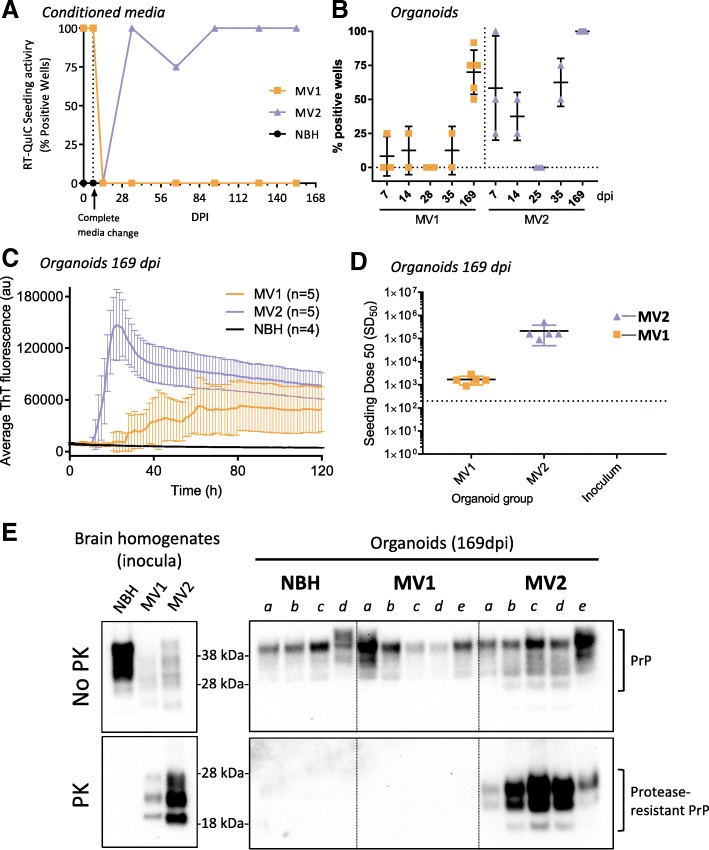
RT-QuIC seeding and protease resistant PrP analysis. **a** Media RT-QuIC monitoring throughout the duration of infection showing the number of replicate wells positive for seeding activity at each sampling time. Each data point represents media taken from the well containing the same three organoids in Fig. 2. Minor tick marks on the x-axis show weekly full media exchanges. **b** Percentage of positive RT-QuIC test wells of organoids taken at various early time points during infection and when harvested at the conclusion on the experiment (169dpi). Each data point represents an individual organoid tested in RT-QuIC with 4–12 replicate wells. Bars show mean and standard deviation of all organoids in each indicated group. **c** RT-QuIC seeding activity of organoid homogenates diluted 10^− 3^ following harvest at 169 dpi. For clarity, every other datapoint was plotted. RT-QuIC traces are displayed as averages from all organoids in each group. Error bars represent standard deviation. **d** SD_50_ values of each organoid harvested at 169 dpi and the original inoculums. Each point represents an individual organoid (or inoculum) with black lines denoting the mean and colored bars the standard deviation. The dotted line represents the threshold of detection below which results are considered negative. **e** Western blots showing PrP and protease-resistant PrP in the original brain homogenate inocula (left plate) and the organoids harvested at 169 dpi (right plate)

RT-QuIC analysis was used to monitor the organoids throughout the incubation period, by assaying culture media for prion seeding activity. One week after the full media exchange out of the inoculum at 7 days post addition (14 dpi), no RT-QuIC seeding activity remained within the media of the MV1 or MV2 organoids. For the NBH and MV1 inoculated organoids, culture media remained RT-QuIC negative for the duration of the study. However, seeding activity became detectable in the media of the organoids receiving the MV2 inoculum at 35 dpi (Fig. 3a).

Over the first few weeks of the infections, several organoids were harvested to determine the uptake of seeding activity from the inoculum and if, or how quickly, organoids could degrade the inoculum to an undetectable level. Organoids harvested at 7 days, the end of the inoculum exposure period, showed seeding activity likely resulting from remaining inoculum. MV1 organoids had less seeding activity uptake from the inocula than MV2, with only a low percentage of wells showing positivity per organoid (Fig. 3b). At 14 days, seeding was detected similarly but by 25–28 dpi all inoculated organoids tested had cleared the original inoculum with no measurable seeding activity remaining in any well. For both inoculations seeding activity had re-appeared at 35 dpi. By comparison organoids harvested at the end of the experiment (169 dpi) showed 50% or greater positive wells for the MV1 inoculated organoids and 100% positive for all MV2 organoids, indicating de novo production of seeding activity as opposed to persistence of the original inoculum within the tissue. Overall, the MV2 inoculated organoids showed greater seeding activity than the MV1 inoculated when harvested at 169 dpi (Fig. 3c). MV2 inoculated organoids had an average seeding SD_50_ of 2.13(±1.63) × 10^5^ per mg of tissue, while the MV1 inoculated organoids had an average SD_50_ of 1.69(±0.70) × 10^3^ per mg of tissue (Fig. 3d) and NBH treated organoids showed no seeding activity (Fig. 3c).

### MV2 infected organoids showed protease-resistant PrP

Protease-resistant PrP is often considered a hallmark of prion disease. Western blotting, following proteinase-K digestion, of the organoid tissue harvested at 169 dpi showed clear protease-resistant PrP banding in all of the MV2 treated organoids with an apparent shift towards a di-glycosylation dominant banding pattern with less unglycosylated PrP (Fig. 3e). Protease-resistant PrP was undetectable by western blot in the MV1 treated organoids, consistent with the lower RT-QuIC seeding activity. PrP^Res^ could not be detected the MV1 infected organoids by western blot even following sodium phosphotungstic acid precipitation (data not shown).

### PrP immunostaining was altered in MV2 inoculated organoids

**Fig. 4:**
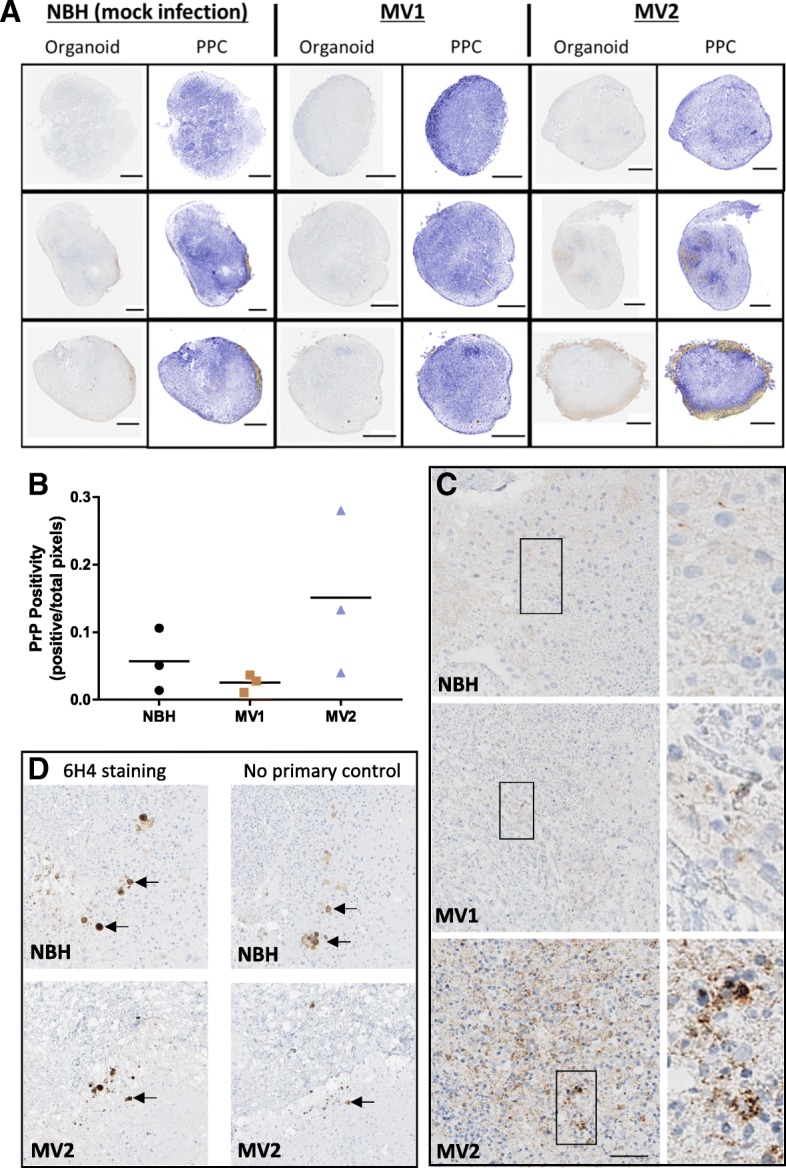
Immunohistochemical staining for PrP in organoids harvested at 169 dpi. **a** Whole organoid images; left panels per condition show 6H4 PrP staining (brown) and right panels showing positive pixel counts (PPC), blue signifies low and yellow-orange denotes high pixel staining intensity. Scale bars = 500 μm. **b** PrP positive pixel counts. Each data point represents an individual organoid with the mean indicated as a line (Kruskal-Wallis, *p* = 0.132). **c** 6H4 PrP staining of organoid interiors. Scale bar = 50 μm and applies to left panels. Right panels are zoomed images of the boxed regions. **d** PrP staining artifacts visible in NBH and MV2 treated organoids. Left panels show 6H4 PrP staining and right panels show no-primary antibody control stains. Arrows indicate artifacts in sequential slices. Scale is as for C

During CJD, prions accumulate within the brain resulting in different immunohistochemical appearances of PrP from the normal protein found in individuals without prion disease. In the cerebral organoids, total PrP immunohistochemical staining was not significantly increased as a result of infection at either 96 dpi (Additional file 1: Figure S1) or 169 dpi (Fig. 4a/b). PrP staining was often seen toward the periphery of the organoid regardless of inoculum (Fig. 4a). However the MV2 organoids showed areas of PrP staining internally. The internal staining of the MV2 inoculated organoids showed areas of course, punctate, granular PrP indicative of a sporadic CJD-like staining pattern, while the MV1 organoids were indistinguishable from controls (Fig. 4c). Some artifactual staining was additionally observed in both the controls and the MV2 stained organoids (Fig. 4d); this was unrelated to pathology as it was visible in the sequential no-primary antibody control sections. These pigments are often found within healthy organoids, visible by eye when observing organoids in culture, and may be retinal pigment or neuromelanin [20]. In the case of the latter, an enhanced pigment intensity due to PrP immunoreactivity would not be unexpected as PrP has been shown to bind to melanin pigments [15].

### Histochemistry showed no differences between sCJD inoculated and control organoids

**Fig. 5:**
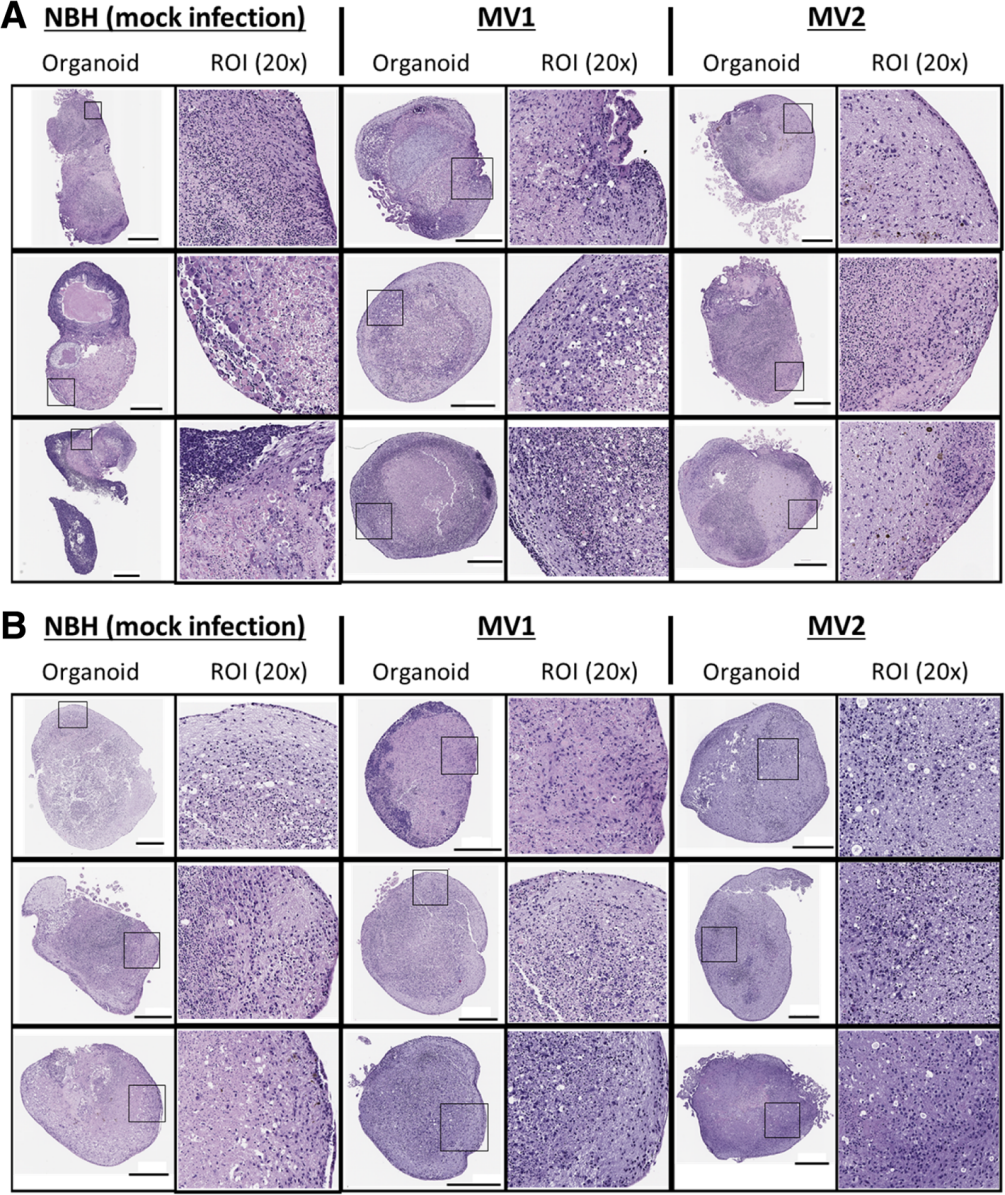
*H&E staining.* Whole organoid and 20× magnification images of H&E stains at **a** 96 dpi and **b** 169 dpi. Regions magnified are high PrP intensity, high astrocyte intensity or both. Scale bar = 500 μm

Vacuolation and astrogliosis are further hallmarks of prion disease. Therefore, this was assessed by hematoxylin and eosin (H&E) histochemistry. Organoids showed diverse regions of organized cells with different staining intensities, consistent with previous descriptions of organoid self-organization [20, 35](Fig. 5a). At 96 dpi, vacuolation was apparent in all three organoids given the MV1 inocula and in two of the three MV2 treated organoids but also in one of three of the NBH treated organoids, indicating that vacuolation occurs from non-prion causes as a normal event within these tissues. At 169 dpi, the NBH treated organoids showed significant vacuolation (Fig. 5b) indicating that this parameter cannot be used as a measure of disease pathology in these organoids.

**Fig. 6:**
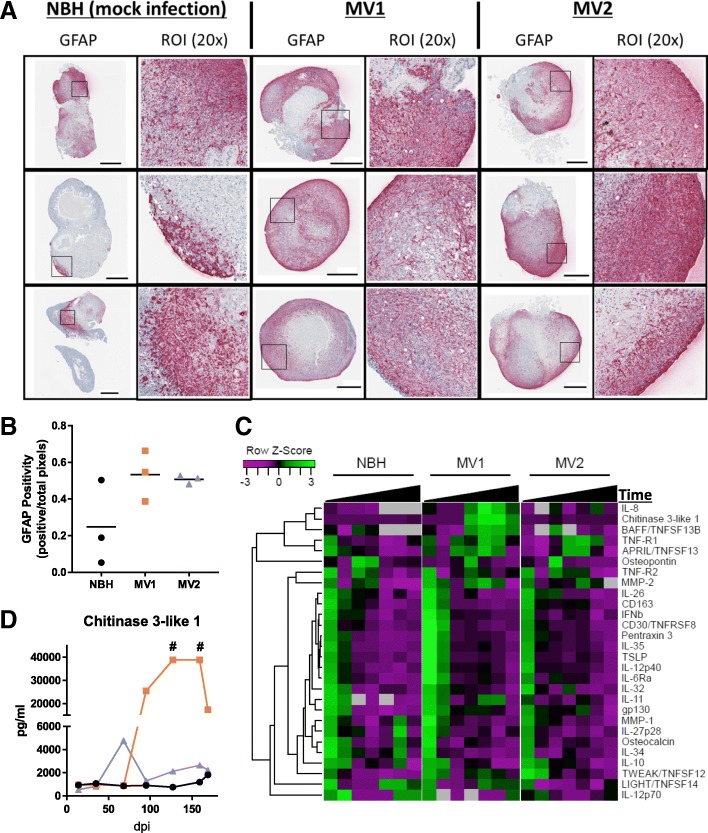
Astrocyte changes during infection. **a** Whole organoid images at 96 dpi; left panels per condition show GFAP staining (pink) and right panels showing positive pixel counts (PPC), blue signifies low and yellow-orange denotes high pixel staining intensity. Scale bars = 500 μm. **b** GFAP positive pixel counts of the 96 dpi whole organoid images. Each data point represents an individual organoid with the mean indicated as a line (Kruskal-Wallis, *p* = 0.196). **c** Heatmap and dendrogram cluster analysis of cytokine detection in culture medium at 14, 35, 68, 95, 127, 159, 169 dpi. Black triangles showing increasing time from inoculation. Data points are generated using media pools from 6 individual organoids. Grey boxes indicate cytokine concentrations below the limits of detection. **d** Concentrations of Chitinase 3-like 1 in culture medium over time. # readings exceeded the accurate range of detection for the assay

Astrogliosis was assessed by detection of glial fibrillary acidic protein (GFAP), an astrocyte marker. At 96 dpi, GFAP staining appeared increased in both the MV1 and MV2 inoculated organoids, however one of the control NBH organoids also had a high density of astrocytes indicating this may not be disease specific but reflect the rate of maturation within the heterogenous organoids (Fig. 6a and b). GFAP staining was highly increased 169 dpi in all organoids including controls (Additional file 1: Figure S2). As GFAP expression is known to increase in normal astrocytes during aging [7, 28], this traditional marker of astrogliosis in prion disease may have limited utility for organoid studies where cultures are aged for long time periods.

An alternative measure of astrocyte activation is variation in cytokine secretion indicative of neuroinflammation. Neuroinflammation is usually mediated by microglia and astrocytes, but, since the organoids lack microglia (microglia differentiate from mesoderm rather than the induced neuroectoderm), changes detected can be attributed primarily to astrocytes. We should note here that while a recent study has shown microglia can differentiate within cerebral organoid cultures from residual mesoderm-derived progenitors [29], no Iba1 positive cell staining (indicative of microglia) was observed in our organoid cultures (data not shown). Whilst microglia are thought to play an important role in prion disease pathogenesis, recent research has shown that they are not necessary for disease progression, appearing to play a protective role against pathogenesis [6], and so we deemed their absence would not hinder disease manifestation within the organoids. For cytokine screening, media pools were collected at 14, 35, 68, 95, 127, 159, and 169 dpi and cluster analysis was used to group cytokines such that the most closely correlated detection patterns are aligned. Most detected cytokines were highest at 14 dpi and then declined in all organoids regardless of inoculum (Fig. 6c). However, a cluster of cytokines with increased expression from 95 dpi was observed in the culture medium from the MV1 inoculated organoids, including IL-8, chitinase 3-like 1, BAFF/TNFSF13B, TNFR1 and APRIL/TNFSF13. The MV2 inoculated organoids showed a similar increase in TNF-R1 and APRIL/TNFSF13 but otherwise showed little change from NBH inoculated organoids. Notably, the increase in chitinase 3-like 1 secretion measured for the MV1 inoculated organoids was almost 30-fold, with two samplings (at 127 and 159 dpi) showing readings in excess of the accurate limit of detection.

## Discussion

Herein, we have demonstrated that iPSC-derived human cerebral organoid cultures can be used to model prion transmission, propagation and limited disease pathology. We show that two different sporadic CJD inocula affect the same underlying organoid line differently, supporting that the prion seed greatly influences the disease phenotype in genetically identical human cells. Our data also indicated that the underlying cell type itself influences the protease-resistant prions that are formed; in all of the MV2 organoids, a shift from the monoglycosylation dominant banding pattern of the sCJD inoculum to diglycosylation dominant indicates that the prions have adapted as they propagated within the organoids.

The MV2 inoculum produced more overt measures of infection in the organoids than the MV1 inoculum, with clear protease-resistant PrP and PrP tissue deposition. The MV1 inoculated organoids generated de novo seeding activity but did not show these traditional hallmarks of prion infection. It is probable that the MV1 PrP seeding the RT-QuIC reaction was close to the limits of detection, and consequently too low to be detected by traditional techniques. Alternatively, the newly synthesized MV1 prions might be protease sensitive. The presence of protease sensitive PrP is a possibility since as much as 90% of the mis-folded PrP in CJD brain tissue has been reported to be protease sensitive [37], and in mouse models of prion infection, PrP mutations that cause accumulation of protease sensitive prions result in a highly neurotoxic species [8]. Preliminary consideration of insoluble PrP within the organoids indicated that it may be present in some MV1 infected organoids (data not shown), therefore the role of protease sensitive PrP warrants further investigation in future studies. Despite the lack of hallmark indicators of PrP infection, the MV1 inoculum demonstrated more influence on health parameters. We can speculate that this could be a property of the MV1 prions; potentially, a more neurotoxic phenotype might correlate with the shorter median clinical course often observed in MV1 sCJD, typically 4–5 months as compared with ~ 11–17 months in MV2 sCJD [17, 33, 36]. However, whether this is related to subtype or an inoculum-specific effect will not be answerable until a greater number of inocula have been screened within this system.

Other factors may have influenced the differences observed between the two inoculas. Prions spread from cell to cell by a number of methods including tunneling nanotubules and exosomal release [12, 41, 47]. Using mouse prion strains it has been shown that the amount of infectivity released through the exosomal pathway is highly strain dependent [1], and this may also be true for the sCJD subtypes. The prions released into the media through the exosomal pathway may be much lower for the MV1 inoculated organoids than the MV2, resulting in a lack of detection within the media and slower propagation of prions through the tissue. Secretion of the MV2 prions into the culture medium may also have aided spread between the organoids in each culture vessel. The lack of detectable seeding activity from the media of the MV1 inoculated organoids is most likely due to released prions being below the threshold of detection.

Cellular heterogeneity may also contribute to the difference between the organoids inoculated with the MV1 and MV2 prions. During the formation and development of the organoids, various cell populations are patterned [43]. Prion subtypes often exhibit different histopathological lesions within different brain regions. The heterogeneity of the organoids may result in a preference for uptake of an inoculum that attacks the specific neuronal populations developed within the organoid. MV1 infection levels may be lower as organoids contain less neurons capable of propagating MV1 prions or because MV1 prions are more toxic to these populations, causing their death before they have propagated new prions. Organoid technology is continuing to progress, such that organoids can be stimulated to differentiate in specific directions, such as dorsal or ventral forebrain and cerebellum [27, 40]. This utility could prove highly valuable in understanding why different prion subtypes preferentially attack specific brain regions. A further exciting development in organoid technologies is the merging of organoids composed of different brain regions and the exchange of cells that results [3, 43]. This could be adapted to provide valuable information on the spreading of prions from cell to cell within the human brain.

While the MV1 uptake and propagation was clearly lower than observed for the MV2, the MV1 infected organoids showed changes in health as a result of exposure to the inoculum, including increased metabolism, LDH release and cytokine release. Chitinase 3-like 1, Chi3L1 or YKL-40, is an inflammatory cytokine primarily produced in adult astrocytes [46], which can induce death of neurons and oligodendrocytes [23, 45]. Chi3L1 has previously been reported to be increased in MM1 and VV2 sCJD brain [24]. MV1 and MM1 sCJD are grouped as the same subtype due to the similarities in pathology of these two diseases [13, 32], therefore the increase in this cytokine in the MV1 inoculated organoids is consistent with expected in vivo changes. The lack of Chi3L1 change in the MV2 organoids is unclear but detection may have increased had these organoids been cultured for longer.

Infection of hu-iPSC derived neuronal cultures has been attempted previously using both ‘normal’ cells and cells carrying the prion Y218N mutation [26]. These infections were unsuccessful, but there may be several reasons that explain the difference from our own study. First, the hu-iPSCs infected in the Y218N study were differentiated as monolayers, which may lose infected cells over serial passages. Second, the authors followed the cultures until 77–79 dpi, but our model had not shown PrP deposition in the tissues at 96 dpi. Therefore, longer incubations may be required to demonstrate infection, which may or may not be possible in the previous model.

Our model demonstrates that cerebral organoids can propagate prion infection from sCJD sources, and that different sCJD subtypes may manifest differently within the same underlying cell. Whilst this latter point is currently being validated, requiring a greater number of inocula to be screened beyond the two tested herein, the capability of organoids differentiated from the same underlying iPSCs to differentiate distinct sCJD subtypes based on their biological properties may be of immense value for understanding the pathologies caused by different human prions.

## Conclusion

Human cerebral organoids are a promising new system for modelling prion disease in cell culture. Organoids take up prion infection, influenced by the prion seed to which they are exposed, and generate new seeding activity. Furthermore, they can produce glycosylated protease-resistant PrP and PrP deposition. We acknowledge that the cerebral organoids have clear limitations; they are highly heterogenous, not vascularized and they may lack non-neuronally derived cells of the brain such as epithelial cells and microglia. Despite these limitations, these cultures represent the closest in vitro cell model to human brain currently available and offer the first human three-dimensional model of prion disease. Their use holds promise for the investigation of different subtype pathologies, for investigation of how prions spread throughout the brain and for trialing putative therapeutics in a human tissue background.

## Additional file

Additional file 1: **Figure S1.** *Whole organoid PrP staining at 96dpi.* Upper panels display whole organoid images; left panels per condition show 6H4 PrP staining (brown) and right panels showing positive pixel counts (PPC), blue signifies low and yellow-orange denotes high pixel staining intensity. Scale bars = 500 μm. Lower graph shows PrP positive pixel counts. Each data point represents an individual organoid with the mean indicated as a line. **Figure S2.** *GFAP staining of organoids.* A. Panels display whole organoid (left) and 20x magnified images of regions of interest (right) of organoids harvested at 169 dpi. B. Positive pixel counts (PPC) at 96 and 169 dpi, blue signifies low and yellow-organe denotes high pixel staining intensity. Scale bars = 500 µm. C. GFAP positive pixel count quantification at 169 dpi. Each data point represents an indevidual organoid with the mean indicated as a line. (DOCX 1790 kb)
