# Supplementary material for: Sporadic Creutzfeldt-Jakob disease prion infection of human cerebral organoids
Source: Acta Neuropathol Commun. 2019 Jun 14;7:12. doi: 10.1186/s40478-019-0742-2 (PMC6567389; doi:10.1186/s40478-019-0742-2)

**Additional File 1**

***Figure S1.*** *Whole organoid PrP staining at 96dpi.* Upper panels display whole organoid images; left panels per condition show 6H4 PrP staining (brown) and right panels showing positive pixel counts (PPC), blue signifies low and yellow-orange denotes high pixel staining intensity. Scale bars = 500 µm. Lower graph shows PrP positive pixel counts. Each data point represents an individual organoid with the mean indicated as a line.


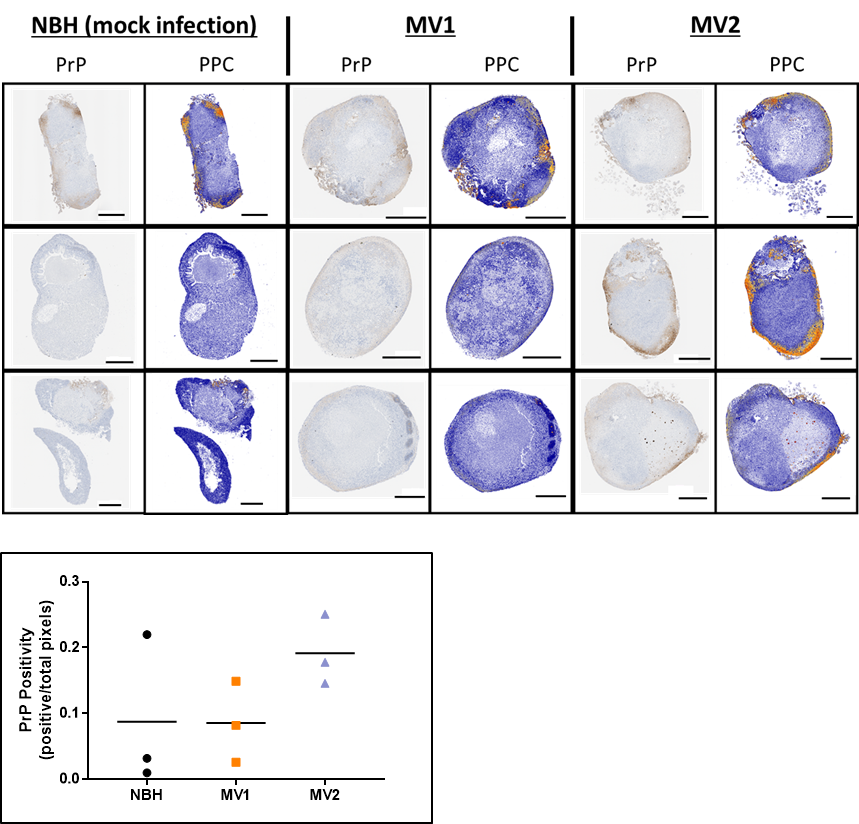


***Figure S2.*** *GFAP staining of organoids.* **A.** Panels display whole organoid (left) and 20× magnified images of regions of interest (right) of organoids harvested at 169 dpi. **B.** Positive pixel counts (PPC) at 96 and 169 dpi, blue signifies low and yellow-orange denotes high pixel staining intensity. Scale bars = 500 µm. **C.** GFAP positive pixel count quantification at 169 dpi. Each data point represents an individual organoid with the mean indicated as a line.


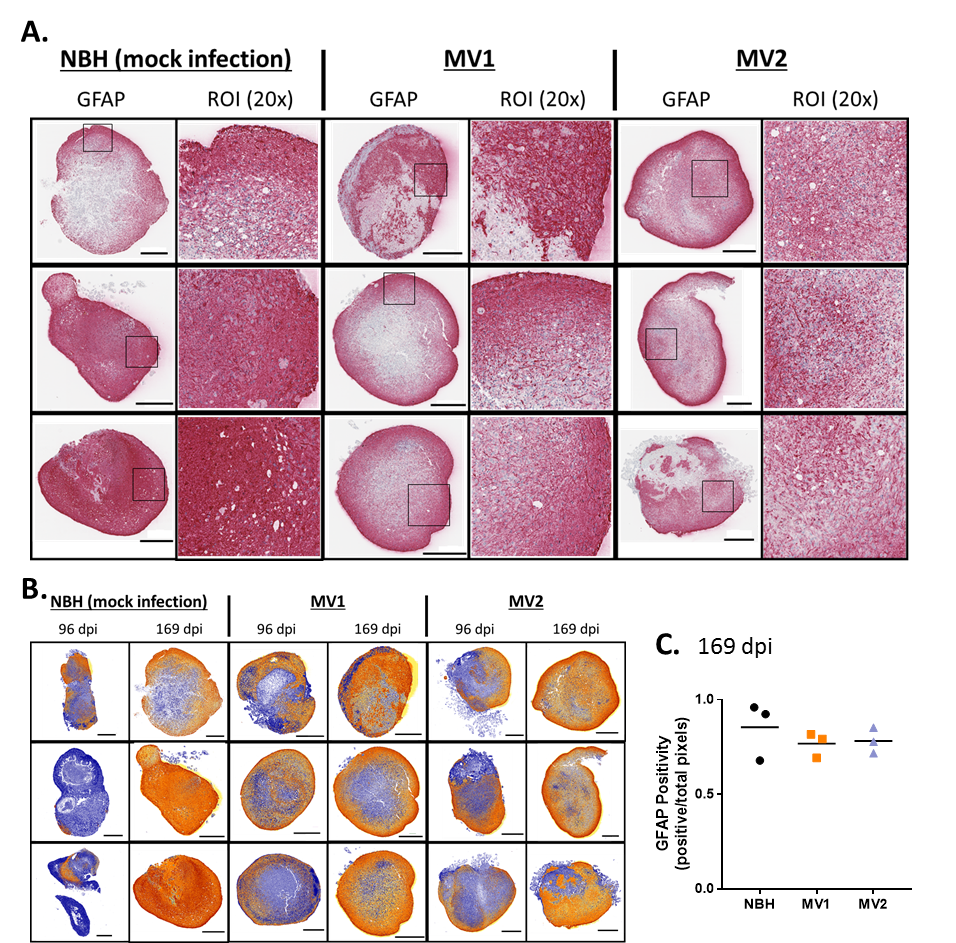

Supplement: Supplementary file 1 — Figure S1. Whole organoid PrP staining at 96dpi. Upper panels display whole organoid images; left panels per condition show 6H4 PrP staining (brown) and right panels showing positive pixel counts (PPC), blue signifies low and yellow-orange denotes high pixel staining intensity. Scale bars = 500 μm. Lower graph shows PrP positive pixel counts. Each data point represents an individual organoid with the mean indicated as a line. Figure S2. GFAP staining of organoids. A. Panels display whole organoid (left) and 20x magnified images of regions of interest (right) of organoids harvested at 169 dpi. B. Positive pixel counts (PPC) at 96 and 169 dpi, blue signifies low and yellow-organe denotes high pixel staining intensity. Scale bars = 500 µm. C. GFAP positive pixel count quantification at 169 dpi. Each data point represents an indevidual organoid with the mean indicated as a line. (DOCX 1790 kb) [file 40478_2019_742_MOESM1_ESM.docx]
